# Bis[μ-*N*-(*tert*-butyl­dimethyl­silyl)-*N*-(pyridin-2-ylmeth­yl)amido]­bis­[methyl­cobalt(II)]

**DOI:** 10.1107/S1600536812032321

**Published:** 2012-08-11

**Authors:** Astrid Malassa, Christine Agthe, Helmar Görls, Matthias Westerhausen

**Affiliations:** a Institut für Anorganische und Analytische Chemie, Friedrich-Schiller-Universität, Jena, Humboldt-Strasse 8, 07743 Jena, Germany

## Abstract

The green title complex, [Co_2_(CH_3_)_2_(C_12_H_21_N_2_Si)_2_], was obtained from bis­{[μ-*N*-*tert*-butyl­dimethyl­silyl-*N*-(pyridin-2-ylmeth­yl)amido]­chloridocobalt(II)} and methyl­lithium in diethyl ether at 195 K *via* a metathesis reaction. The dimeric cobalt(II) complex exhibits a crystallographic center of inversion in the middle of the Co_2_N_2_ ring (average Co—N = 2.050 Å). The Co^II^ atom shows a distorted tetra­hedral coordination sphere. The exocyclic Co—N bond length to the pyridyl group shows a similar value of 2.045 (4) Å. The exocyclic methyl group has a rather long Co—C bond length of 2.019 (5) Å.

## Related literature

The metathetical conversion of a cobalt chloride functionality into a methyl cobalt fragment *via* the reaction with methyllithium was reported earlier for tetra-coordinate cobalt(II) complexes bound to three additional aza-bases, see: Au-Yeung *et al.* (2007 ▶); Bowman *et al.* (2010 ▶); Humphries *et al.* (2005 ▶); Kleigrewe *et al.* (2005 ▶), Wallenhorst *et al.* (2008 ▶). The synthesis of dialkyl cobalt complexes succeeds starting from hexa-coordinate [(*L*)_4_CoCl_2_] with *L* being a pyridyl base, see: Milani *et al.* (2003 ▶); Zhu *et al.* (2010 ▶). The coordination number of the final cobalt(II) complexes depends on intra­molecular steric strain yielding hexa-coordinate [(bpy)_2_CoMe_2_] (bpy = 2,2′-bipyridine) and tetra-coordinate [(py)_2_Co*R*_2_] (*R* = CH_2_C(Me_2_)Ph). The formation of *para*-tolyl­cobalt complexes was reported by Zhu & Budzelaar (2010 ▶) who proposed a radical mechanism.
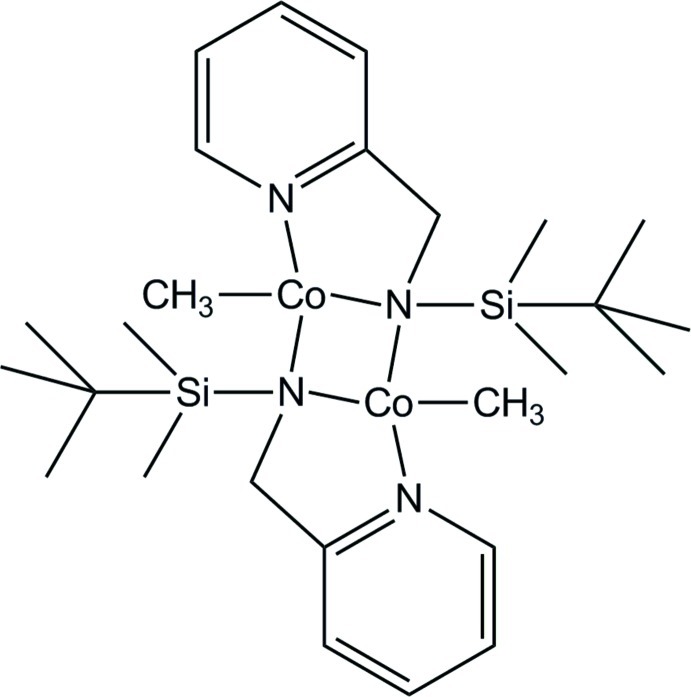


## Experimental

### 

#### Crystal data

[Co_2_(CH_3_)_2_(C_12_H_21_N_2_Si)_2_]
*M* *_r_* = 590.72
Triclinic, 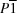

*a* = 8.4751 (8) Å
*b* = 9.8055 (12) Å
*c* = 10.6130 (6) Å
α = 72.837 (6)°
β = 83.450 (6)°
γ = 69.216 (6)°
*V* = 787.81 (13) Å^3^
*Z* = 1
Mo *K*α radiation
μ = 1.15 mm^−1^
*T* = 183 K
0.06 × 0.06 × 0.04 mm

#### Data collection

Nonius KappaCCD diffractometer
5417 measured reflections
3551 independent reflections
1685 reflections with *I* > 2σ(*I*)
*R*_int_ = 0.074

#### Refinement

*R*[*F* ^2^ > 2σ(*F* ^2^)] = 0.061
*wR*(*F* ^2^) = 0.129
*S* = 0.92
3551 reflections
160 parameters
H-atom parameters constrained
Δρ_max_ = 0.39 e Å^−3^
Δρ_min_ = −0.39 e Å^−3^

### 

Data collection: *COLLECT* (Nonius, 1998 ▶); cell refinement: *DENZO* (Otwinowski & Minor, 1997 ▶); data reduction: *DENZO*; program(s) used to solve structure: *SHELXS97* (Sheldrick, 2008 ▶); program(s) used to refine structure: *SHELXL97* (Sheldrick, 2008 ▶); molecular graphics: *SHELXTL/PC* (Sheldrick, 2008 ▶); software used to prepare material for publication: *SHELXL97*.

## Supplementary Material

Crystal structure: contains datablock(s) I, global. DOI: 10.1107/S1600536812032321/im2393sup1.cif

Structure factors: contains datablock(s) I. DOI: 10.1107/S1600536812032321/im2393Isup2.hkl

Additional supplementary materials:  crystallographic information; 3D view; checkCIF report

## Figures and Tables

**Fig. 1.**
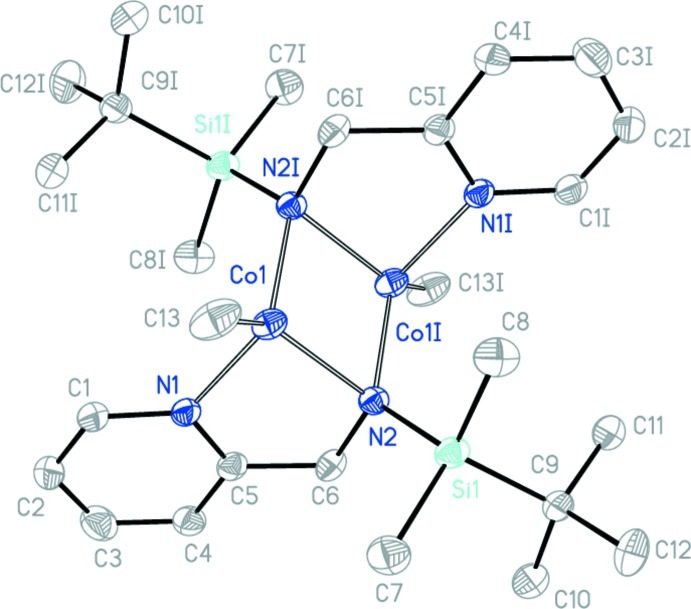
Molecular structure and numbering scheme of the title compound 1; Displacement ellipsoids are at the 40% probability level. H atoms are neglected for clarity reasons. Symmetry-related atoms are marked with the letter i [symmetry code: (i) -x + 1, -y + 1, -z + 1].

**Fig. 2.**
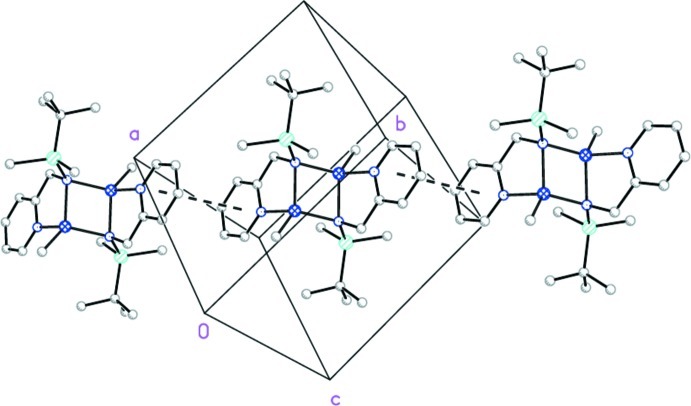
Packing of the molecules by short ring-interactions (distance between the centroids of the aromatic rings 3.689 (3) Å).

## supplementary crystallographic information

### Comment

Au-Yeung *et al.* (2007) performed a metathetical ligand substitution reaction at (tmeda)cobalt(II) 2,6-dimethylphenyl-*N*-trimethylsilylamide chloride (tmeda = tetramethylethylenediamine) with methyllithium in toluene. Whereas in this complex the cobalt(II) adopts a distorted tetrahedral coordination sphere, severe distortions were observed using tridentate aza-Lewis bases (Bowman *et al.*, 2010; Humphries *et al.*, 2005; Kleigrewe *et al.*, 2005; Wallenhorst *et al.*, 2008). Treatment of tetrakis(pyridine)cobalt(II) dichloride with trimethylsilylmethyllithium or 2-methyl-2-phenylpropyllithium in n-pentane yielded [(py)_2_Co*R*_2_] (*R* = CH_2_SiMe_3_)_2_, CH_2_C(Me_2_)Ph), respectively, with tetra-coordinate cobalt centers (Zhu *et al.*, 2010). Less bulky methyl groups allowed the formation of [(bpy)_2_CoMe_2_] with a hexa-coordinate cobalt atom in a slightly distorted octahedral environment (Milani *et al.*, 2003). Contrary to these procedures, a radical mechanism was discussed by Zhu & Budzelaar (2010) for the formation of *para*-tolyl-cobalt complexes. Whereas all of these cobalt(II) complexes represent mononuclear derivatives, the reaction of bis[*N*-(pyidin-2-ylmethyl)-*N*-(*tert*-butyldimethylsilyl)amido cobalt(II) chloride] with methyllithium in tetrahydrofuran (THF) yielded the centrosymmetric dinuclear title compound 1 with a central planar Co_2_N_2_ ring.

### Experimental

Bis{chlorido-[*N*-(pyidin-2-ylmethyl)-*N*-(*tert*-butyldimethylsilyl)amido]cobalt(II)} (0.84 g, 1.32 mmol) was dissolved in 15 ml of THF and this solution cooled to -78 °C. Then 1.7 ml (2,72 mmol) of a 1,6*M* methyllithium solution in diethyl ether was added dropwise. A brown reaction solution formed which was warmed to ambient temperature and stirred for an additional hour. Thereafter all volatile materials were removed and the residue dried *in vacuo*. This residue was extracted with 15 ml of n-hexane. The volume of this solution was reduced to third of the original volume and cooled to -20 °C. Within several hours green rod-like crystals of 1 precipitated. Yield: 0.21 g (0.36 mmol, 27%).

### Refinement

All hydrogen atoms were calculated to idealized positions with C–H distances of 0.98 (methyl), 0.99 (methylene) and 0.95 (phenyl) Å, and were refined with 1.2 times (1.5 for all methyl groups) the isotropic displacement parameter of the corresponding carbon atom. All methyl groups were allowed to rotate but not to tip.

### Crystal data

**Table:**

[Co_2_(CH_3_)_2_(C_12_H_21_N_2_Si)_2_]	*Z* = 1
*M**_r_* = 590.72	*F*(000) = 314
Triclinic, *P*1	*D*_x_ = 1.245 Mg m^−^^3^
Hall symbol: -P 1	Mo *K*α radiation, λ = 0.71073 Å
*a* = 8.4751 (8) Å	Cell parameters from 5417 reflections
*b* = 9.8055 (12) Å	θ = 3.3–27.5°
*c* = 10.6130 (6) Å	µ = 1.15 mm^−^^1^
α = 72.837 (6)°	*T* = 183 K
β = 83.450 (6)°	Prism, green
γ = 69.216 (6)°	0.06 × 0.06 × 0.04 mm
*V* = 787.81 (13) Å^3^	

### Data collection

**Table:**

Nonius KappaCCD diffractometer	1685 reflections with *I* > 2σ(*I*)
Radiation source: fine-focus sealed tube	*R*_int_ = 0.074
Graphite monochromator	θ_max_ = 27.5°, θ_min_ = 3.3°
phi– + ω–scan	*h* = −10→9
5417 measured reflections	*k* = −10→12
3551 independent reflections	*l* = −13→13

### Refinement

**Table:**

Refinement on *F*^2^	Primary atom site location: structure-invariant direct methods
Least-squares matrix: full	Secondary atom site location: difference Fourier map
*R*[*F*^2^ > 2σ(*F*^2^)] = 0.061	Hydrogen site location: inferred from neighbouring sites
*wR*(*F*^2^) = 0.129	H-atom parameters constrained
*S* = 0.92	*w* = 1/[σ^2^(*F*_o_^2^) + (0.0369*P*)^2^] where *P* = (*F*_o_^2^ + 2*F*_c_^2^)/3
3551 reflections	(Δ/σ)_max_ < 0.001
160 parameters	Δρ_max_ = 0.39 e Å^−^^3^
0 restraints	Δρ_min_ = −0.39 e Å^−^^3^

### Special details

**Table:**

Experimental. *IR* (in Nujol between KBr windows, cm^-1^): = 1715 w, 1583 m, 1273 m, 1244 s, 1146 m, 1080 m, 1036 m, 1008 m, 889 m, 828 s, 770 m, 736 m. *MS* (DEI, *rel*. intensity in brackets): m/*z* = 501 ([*M* - CoMe_2_]^+^, 11%), 165 ([Pyr-CH_2_-NHSiMe_2_]^+^, 100%). *Elemental analysis* (C_26_H_48_Co_2_N_4_Si_2_, 590,72): calcd.: C 52.86, H 8.19, N 9.48; found: C 49.47, H 7.70, N 9.03 (the rather large deviations are caused by extreme sensitivity of the complex towards moisture and air; the low carbon value is a consequence of carbide and carbonate formation despite the fact that V_2_O_5_ was added prior to combustion).
Geometry. All e.s.d.'s (except the e.s.d. in the dihedral angle between two l.s. planes) are estimated using the full covariance matrix. The cell e.s.d.'s are taken into account individually in the estimation of e.s.d.'s in distances, angles and torsion angles; correlations between e.s.d.'s in cell parameters are only used when they are defined by crystal symmetry. An approximate (isotropic) treatment of cell e.s.d.'s is used for estimating e.s.d.'s involving l.s. planes.
Refinement. Refinement of *F*^2^ against ALL reflections. The weighted *R*-factor *wR* and goodness of fit *S* are based on *F*^2^, conventional *R*-factors *R* are based on *F*, with *F* set to zero for negative *F*^2^. The threshold expression of *F*^2^ > σ(*F*^2^) is used only for calculating *R*-factors(gt) *etc*. and is not relevant to the choice of reflections for refinement. *R*-factors based on *F*^2^ are statistically about twice as large as those based on *F*, and *R*- factors based on ALL data will be even larger.

### Fractional atomic coordinates and isotropic or equivalent isotropic displacement parameters (Å^2^)

**Table:**

	*x*	*y*	*z*	*U*_iso_*/*U*_eq_	
Co1	0.50875 (8)	0.62393 (8)	0.52394 (5)	0.0360 (2)	
Si1	0.36965 (16)	0.43232 (17)	0.77488 (11)	0.0373 (4)	
N1	0.2891 (5)	0.7874 (4)	0.4452 (3)	0.0342 (10)	
N2	0.3562 (4)	0.4948 (4)	0.6037 (3)	0.0306 (9)	
C1	0.2668 (7)	0.9328 (6)	0.3756 (4)	0.0466 (14)	
H1A	0.3599	0.9681	0.3636	0.056*	
C2	0.1139 (7)	1.0311 (6)	0.3217 (4)	0.0524 (15)	
H2A	0.1017	1.1325	0.2732	0.063*	
C3	−0.0218 (7)	0.9803 (7)	0.3391 (5)	0.0570 (16)	
H3A	−0.1275	1.0452	0.3003	0.068*	
C4	−0.0012 (6)	0.8329 (6)	0.4142 (4)	0.0429 (13)	
H4A	−0.0938	0.7967	0.4294	0.052*	
C5	0.1560 (6)	0.7389 (6)	0.4668 (4)	0.0349 (12)	
C6	0.1808 (5)	0.5827 (6)	0.5525 (4)	0.0387 (12)	
H6A	0.1501	0.5256	0.5020	0.046*	
H6B	0.1020	0.5892	0.6286	0.046*	
C7	0.2682 (6)	0.5989 (6)	0.8446 (4)	0.0529 (15)	
H7A	0.3243	0.6742	0.8080	0.079*	
H7B	0.2788	0.5648	0.9408	0.079*	
H7C	0.1485	0.6445	0.8213	0.079*	
C8	0.5975 (6)	0.3468 (6)	0.8222 (4)	0.0504 (15)	
H8A	0.6571	0.4165	0.7746	0.076*	
H8B	0.6479	0.2504	0.7993	0.076*	
H8C	0.6067	0.3288	0.9174	0.076*	
C9	0.2621 (6)	0.2877 (6)	0.8564 (4)	0.0430 (13)	
C10	0.0704 (6)	0.3488 (6)	0.8314 (5)	0.0594 (16)	
H10A	0.0208	0.2719	0.8808	0.089*	
H10B	0.0505	0.3722	0.7370	0.089*	
H10C	0.0180	0.4411	0.8606	0.089*	
C11	0.3389 (6)	0.1452 (6)	0.8085 (4)	0.0502 (14)	
H11A	0.2790	0.0739	0.8499	0.075*	
H11B	0.4584	0.0976	0.8324	0.075*	
H11C	0.3288	0.1728	0.7125	0.075*	
C12	0.2873 (7)	0.2393 (7)	1.0077 (4)	0.0700 (19)	
H12A	0.2415	0.1575	1.0491	0.105*	
H12B	0.2284	0.3263	1.0430	0.105*	
H12C	0.4080	0.2034	1.0267	0.105*	
C13	0.6200 (6)	0.7259 (7)	0.6082 (4)	0.0580 (16)	
H13A	0.6269	0.8189	0.5442	0.087*	
H13B	0.7339	0.6571	0.6363	0.087*	
H13C	0.5532	0.7510	0.6850	0.087*	

### Atomic displacement parameters (Å^2^)

**Table:**

	*U*^11^	*U*^22^	*U*^33^	*U*^12^	*U*^13^	*U*^23^
Co1	0.0333 (4)	0.0450 (5)	0.0363 (4)	−0.0195 (3)	0.0038 (3)	−0.0147 (3)
Si1	0.0355 (8)	0.0459 (10)	0.0285 (7)	−0.0122 (8)	0.0012 (5)	−0.0098 (6)
N1	0.039 (2)	0.033 (3)	0.033 (2)	−0.015 (2)	0.0103 (16)	−0.0130 (19)
N2	0.026 (2)	0.036 (3)	0.0285 (17)	−0.009 (2)	−0.0019 (15)	−0.0068 (16)
C1	0.051 (4)	0.038 (4)	0.050 (3)	−0.017 (3)	0.020 (3)	−0.015 (3)
C2	0.059 (4)	0.037 (4)	0.048 (3)	−0.010 (3)	0.011 (3)	−0.004 (3)
C3	0.051 (4)	0.050 (4)	0.054 (3)	0.000 (3)	−0.005 (3)	−0.010 (3)
C4	0.032 (3)	0.045 (4)	0.046 (3)	−0.009 (3)	0.001 (2)	−0.010 (3)
C5	0.037 (3)	0.041 (3)	0.024 (2)	−0.012 (3)	0.0019 (19)	−0.008 (2)
C6	0.038 (3)	0.044 (4)	0.037 (2)	−0.022 (3)	0.003 (2)	−0.007 (2)
C7	0.061 (4)	0.057 (4)	0.045 (3)	−0.018 (3)	0.003 (2)	−0.024 (3)
C8	0.045 (3)	0.070 (4)	0.038 (3)	−0.014 (3)	−0.007 (2)	−0.020 (3)
C9	0.044 (3)	0.042 (4)	0.029 (2)	−0.005 (3)	0.005 (2)	−0.003 (2)
C10	0.050 (4)	0.054 (4)	0.068 (3)	−0.022 (3)	0.016 (3)	−0.009 (3)
C11	0.048 (3)	0.042 (4)	0.056 (3)	−0.018 (3)	0.010 (2)	−0.007 (3)
C12	0.084 (4)	0.064 (5)	0.040 (3)	−0.019 (4)	0.001 (3)	0.009 (3)
C13	0.055 (4)	0.089 (5)	0.055 (3)	−0.047 (4)	0.013 (3)	−0.034 (3)

### Geometric parameters (Å, º)

**Table:**

Co1—C13	2.019 (5)	C6—H6B	0.9900
Co1—N2^i^	2.032 (3)	C7—H7A	0.9800
Co1—N1	2.045 (4)	C7—H7B	0.9800
Co1—N2	2.067 (4)	C7—H7C	0.9800
Co1—Co1^i^	2.6812 (14)	C8—H8A	0.9800
Si1—N2	1.741 (3)	C8—H8B	0.9800
Si1—C8	1.873 (4)	C8—H8C	0.9800
Si1—C7	1.877 (5)	C9—C11	1.528 (7)
Si1—C9	1.898 (5)	C9—C10	1.544 (6)
N1—C5	1.345 (5)	C9—C12	1.551 (6)
N1—C1	1.354 (6)	C10—H10A	0.9800
N2—C6	1.499 (5)	C10—H10B	0.9800
N2—Co1^i^	2.032 (3)	C10—H10C	0.9800
C1—C2	1.376 (6)	C11—H11A	0.9800
C1—H1A	0.9500	C11—H11B	0.9800
C2—C3	1.381 (7)	C11—H11C	0.9800
C2—H2A	0.9500	C12—H12A	0.9800
C3—C4	1.388 (7)	C12—H12B	0.9800
C3—H3A	0.9500	C12—H12C	0.9800
C4—C5	1.390 (6)	C13—H13A	0.9800
C4—H4A	0.9500	C13—H13B	0.9800
C5—C6	1.487 (6)	C13—H13C	0.9800
C6—H6A	0.9900		
			
C13—Co1—N2^i^	119.40 (17)	N2—C6—H6B	108.5
C13—Co1—N1	105.8 (2)	H6A—C6—H6B	107.5
N2^i^—Co1—N1	112.97 (13)	Si1—C7—H7A	109.5
C13—Co1—N2	130.97 (16)	Si1—C7—H7B	109.5
N2^i^—Co1—N2	98.30 (12)	H7A—C7—H7B	109.5
N1—Co1—N2	84.19 (15)	Si1—C7—H7C	109.5
C13—Co1—Co1^i^	151.34 (17)	H7A—C7—H7C	109.5
N2^i^—Co1—Co1^i^	49.71 (10)	H7B—C7—H7C	109.5
N1—Co1—Co1^i^	102.57 (11)	Si1—C8—H8A	109.5
N2—Co1—Co1^i^	48.59 (10)	Si1—C8—H8B	109.5
N2—Si1—C8	108.95 (18)	H8A—C8—H8B	109.5
N2—Si1—C7	109.2 (2)	Si1—C8—H8C	109.5
C8—Si1—C7	108.4 (2)	H8A—C8—H8C	109.5
N2—Si1—C9	114.74 (19)	H8B—C8—H8C	109.5
C8—Si1—C9	108.4 (2)	C11—C9—C10	107.8 (4)
C7—Si1—C9	107.0 (2)	C11—C9—C12	107.5 (4)
C5—N1—C1	119.0 (4)	C10—C9—C12	107.5 (4)
C5—N1—Co1	113.7 (3)	C11—C9—Si1	111.1 (3)
C1—N1—Co1	127.3 (3)	C10—C9—Si1	113.3 (3)
C6—N2—Si1	114.5 (2)	C12—C9—Si1	109.4 (3)
C6—N2—Co1^i^	109.3 (2)	C9—C10—H10A	109.5
Si1—N2—Co1^i^	125.9 (2)	C9—C10—H10B	109.5
C6—N2—Co1	108.8 (3)	H10A—C10—H10B	109.5
Si1—N2—Co1	111.27 (17)	C9—C10—H10C	109.5
Co1^i^—N2—Co1	81.70 (12)	H10A—C10—H10C	109.5
N1—C1—C2	122.1 (5)	H10B—C10—H10C	109.5
N1—C1—H1A	118.9	C9—C11—H11A	109.5
C2—C1—H1A	118.9	C9—C11—H11B	109.5
C1—C2—C3	119.1 (5)	H11A—C11—H11B	109.5
C1—C2—H2A	120.4	C9—C11—H11C	109.5
C3—C2—H2A	120.4	H11A—C11—H11C	109.5
C2—C3—C4	119.0 (5)	H11B—C11—H11C	109.5
C2—C3—H3A	120.5	C9—C12—H12A	109.5
C4—C3—H3A	120.5	C9—C12—H12B	109.5
C3—C4—C5	119.4 (5)	H12A—C12—H12B	109.5
C3—C4—H4A	120.3	C9—C12—H12C	109.5
C5—C4—H4A	120.3	H12A—C12—H12C	109.5
N1—C5—C4	121.3 (4)	H12B—C12—H12C	109.5
N1—C5—C6	117.8 (4)	Co1—C13—H13A	109.5
C4—C5—C6	120.8 (4)	Co1—C13—H13B	109.5
C5—C6—N2	115.1 (4)	H13A—C13—H13B	109.5
C5—C6—H6A	108.5	Co1—C13—H13C	109.5
N2—C6—H6A	108.5	H13A—C13—H13C	109.5
C5—C6—H6B	108.5	H13B—C13—H13C	109.5

Symmetry code: (i) −*x*+1, −*y*+1, −*z*+1.
